# Multiple Regression Analysis of Ultrasound and Clinical Features for Quantitative Evaluation of Tubal Pregnancy Rupture

**DOI:** 10.1002/jum.70057

**Published:** 2025-09-16

**Authors:** Shuang Gui, Xiao‐qing Liu, Xiao‐hui Hu, Meng‐sen Li, Hong‐Zhou

**Affiliations:** ^1^ Shanghai Ninth People's Hospital Shanghai Jiaotong University School of Medicine Shanghai China

**Keywords:** multiple regression analysis, tubal pregnancy rupture, ultrasound and clinical features

## Abstract

The aim is to search for quantitative indicators of ultrasound and clinical features that suggest tubal pregnancy rupture, and to identify independent risk factors for tubal pregnancy rupture through multiple regression analysis. Retrospective analysis of 166 cases of tubal pregnancy was confirmed by laparoscopy, including 97 cases of unruptured type and 69 cases of ruptured type. Compare the ultrasound and clinical features of the 2 groups to identify quantitative indicators of tubal pregnancy rupture. Ultrasound features include: uterine position (anterior or posterior) and endometrial thickness, location of ectopic pregnancy (right or left), size, morphology, internal echoes, boundaries, and pelvic fluid accumulation. Clinical features include: age, number of days of menopause, abdominal pain and vaginal bleeding, intrauterine device, history of ectopic pregnancy and pelvic inflammatory disease, number of pregnancies and miscarriages, and preoperative β‐human chorionic gonadotropin (β‐HCG) value. Establish receiver operating characteristic (ROC) curves to determine the diagnostic efficacy of various ultrasound and clinical features for tubal pregnancy rupture and the optimal threshold for predicting the cause of rupture. Through multiple logistic regression analysis, identify the risk factors for tubal pregnancy rupture. The ultrasound features of unruptured tubal pregnancy (UNRTP) were: in 20 out of 97 cases, a mixed echo like a gestational sac could be seen in the attachment area, with clear boundaries and partial presence of yolk sac and embryo inside. In 50 out of 97 cases, there was not much pelvic fluid accumulation (<15 mm by ultrasound). Ruptured tubal pregnancy (RTP) ultrasound features were: large mixed echo mass in the attachment area, without obvious boundaries, with chaotic internal echoes, and a large amount of pelvic fluid accumulation. Univariate analysis showed that there was no difference in terms of uterine position, endometrial thickness, and mass location between the 2 groups (*P* > .05), but the RTP group had a larger maximum mass diameter, unclear boundaries, and more pelvic fluid accumulation (*P* < .05). Clinical characteristics: There was no difference in terms of age, vaginal bleeding, intrauterine device, history of ectopic pregnancy, number of pregnancies, history of miscarriage, and surgical methods (*P* > .05), but in the RTP group, there were more cases of abdominal pain, pelvic inflammatory disease, high preoperative β‐HCG (*P* < .05). The ROC curve showed that the maximum diameter of the mass, unclear boundaries, pelvic fluid accumulation, abdominal pain, preoperative β‐HCG, AUC are 0.741, 0.726, 0.752, 0.897, 0.585, 0.631 (all *P* < .05), which could be used to evaluate tubal pregnancy rupture. If the AUC of pelvic inflammatory disease is 0.585 (*P* > .05), it could not be used to evaluate tubal rupture. The cut‐off values showed that the maximum diameter of the mass was >36.5 mm, the pelvic fluid volume measured by ultrasound was >34.5 mm, preoperative HCG > 3094.5 U/L, indicating the possibility of tube pregnancy rupture. Multiple logistic regression analysis showed that the accumulation of pelvic fluid measured by ultrasound and preoperative β‐HCG were independent risk factors for tubal rupture (*P* < .05). The accumulation of pelvic fluid measured by ultrasound and preoperative β‐HCG was independent risk factors for ruptured tubal pregnancy.

**Abbreviations**
ROC: receiver operating characteristic
RTP: ruptured tubal pregnancy
UNRTP: unruptured tubal pregnancy

Ectopic pregnancy refers to the implantation of fertilized eggs in any part outside the uterine cavity, with an incidence of approximately 1–5%, it accounted for 4% of pregnancy‐related death,^1^ including tubes, ovaries, scars in the lower segment of the uterus, cervix, and abdominal cavity, with 95% occurring in the fallopian tubes. This article studied tube pregnancy. When tubal pregnancy persists and the activity of the villi is high without timely diagnosis, the villi can erode the muscular and serous layers of the fallopian tubes, causing rupture, bleeding, abdominal pain, and seriously endangering the safety of women of childbearing age. Therefore, early diagnosis is extremely important. Previous reports^2^, ^3^ had reported that major risk factors for tubal pregnancy include inflammatory diseases of the tubes, placement of intrauterine devices, number of miscarriages, history of ectopic pregnancy and tubal sterilization, older age and smoking. The treatment methods include conservative treatment with intramuscular injection of methotrexate and surgical treatment.^4^, ^5^ For ruptured tubal pregnancy, the diseased tubal must be removed surgically to avoid massive bleeding and endanger the patient's life safety.^6^ Ultrasound had become the primary and important examination method for diagnosing ectopic pregnancy with its advantages of simplicity, convenience, and high reproducibility. Seventy‐five percent of tubal pregnancies can be detected by transvaginal ultrasonography.^6^, ^7^

The purpose of this study is to quantitatively evaluate and summarize the ultrasound and clinical features that suggest tubal pregnancy rupture, obtain the ultrasound and clinical experience that guides clinical doctors to handle it in a timely manner, and ensure the safety of patients' lives.

## Method

### Research Object

Retrospective analysis of 166 cases of ectopic pregnancy diagnosed by surgery at Shanghai Ninth People's Hospital and Obstetrics and Gynecology Hospital of Tongji University from December 2018 to December 2023, including 97 cases of unruptured type and 69 cases of ruptured type. Inclusion criteria: Patients aged 20–45 years who can actively cooperate with examinations, surgical treatment, and pathological diagnosis. Exclusion criteria: conservative treatment and lost to follow‐up patients.

### Methods and Instruments

#### Clinical Data

Age days of menopause, abdominal pain and vaginal bleeding, intrauterine device, history of ectopic pregnancy and pelvic inflammatory disease, number of pregnancies and miscarriages, preoperative β‐human chorionic gonadotropin (β‐HCG) value.

#### Ultrasound Examination Methods and Analysis Content

Using GE Voluson E8 and SAMSUNG‐WS80A ultrasound diagnostic instruments, with intracavity probes and frequencies ranging from 5.0 to 9.0 MHz; the frequency of the transabdominal probe is between 2.0 and 5.0 MHz. Ultrasound examination was performed at the bladder lithotomy site. Vaginal examination required emptying the bladder, while abdominal examination required moderate filling of the bladder. The ultrasound images and data were completed by 2 senior ultrasound physicians in the 5th grade or above. Firstly, perform ultrasound examination on the patient's uterus, bilateral ovaries, and adnexa area, with a focus on observing the implantation position, size, shape, boundary, internal echo, and presence of yolk sac or embryo in the adnexa area.

### Operation

The unruptured and ruptured groups underwent laparoscopic salpingectomy.

### Statistical Methods

Using SPSS 23.0 software, with quantitative data expressed as *x* ± *s* and comparison using *t*‐test; count data were presented as examples or rates, using a chi‐squared test. Drawed, receiver operating characteristic (ROC) curves to analyze the efficacy and optimal threshold of various ultrasound features and clinical indicators in evaluating tubal pregnancy rupture. Youden index was used to select the cut‐off values, Youden index = sensitivity+ specificity‐1, high sensitivity was to avoid missed diagnosis, while high specificity was to avoid misdiagnosis. Use multivariate logistic regression analyzed to identify the risk factors for tubal rupture, with *P* < .05 indicating statistical significance.

### Ethics

This study was approved by the hospital ethics committee (ethics number: KS25234) and the patients provided informed consent.

## Result

### Ultrasound Features of 2 Groups

**Figure 1:**
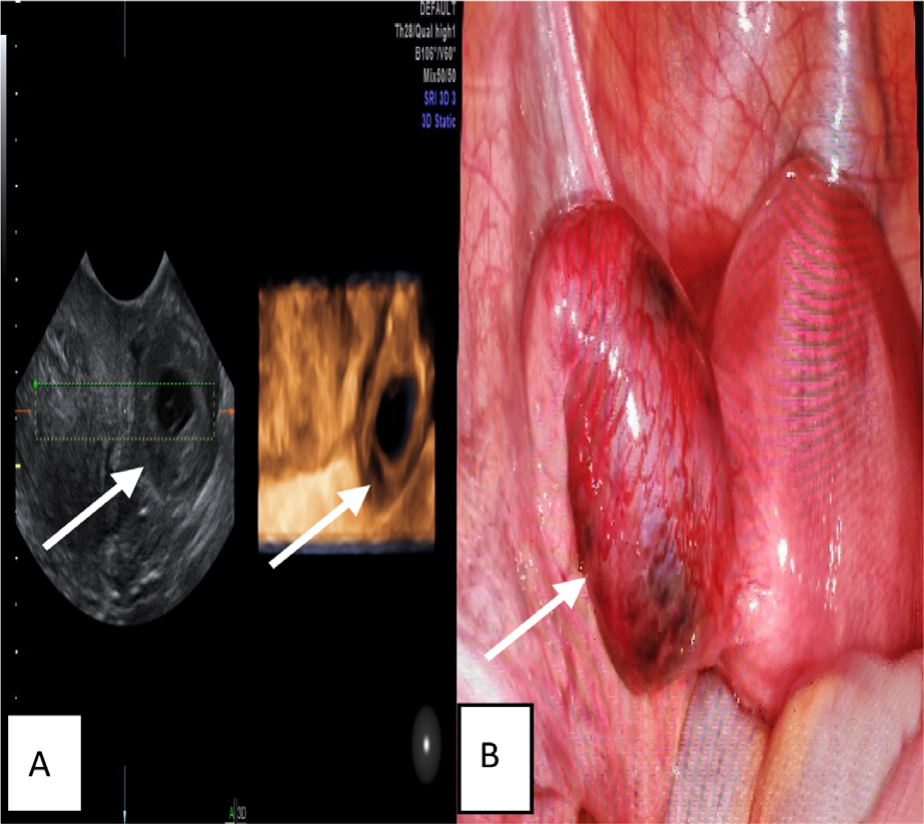
A 32‐year‐old women with UNRTP. **A**, Transverse sonogram showing a mix echo mass in the left tube. **B**, Intraoperative photo displaying thickening of the left fallopian tube, with no visible rupture on the surface.

**Figure 2:**
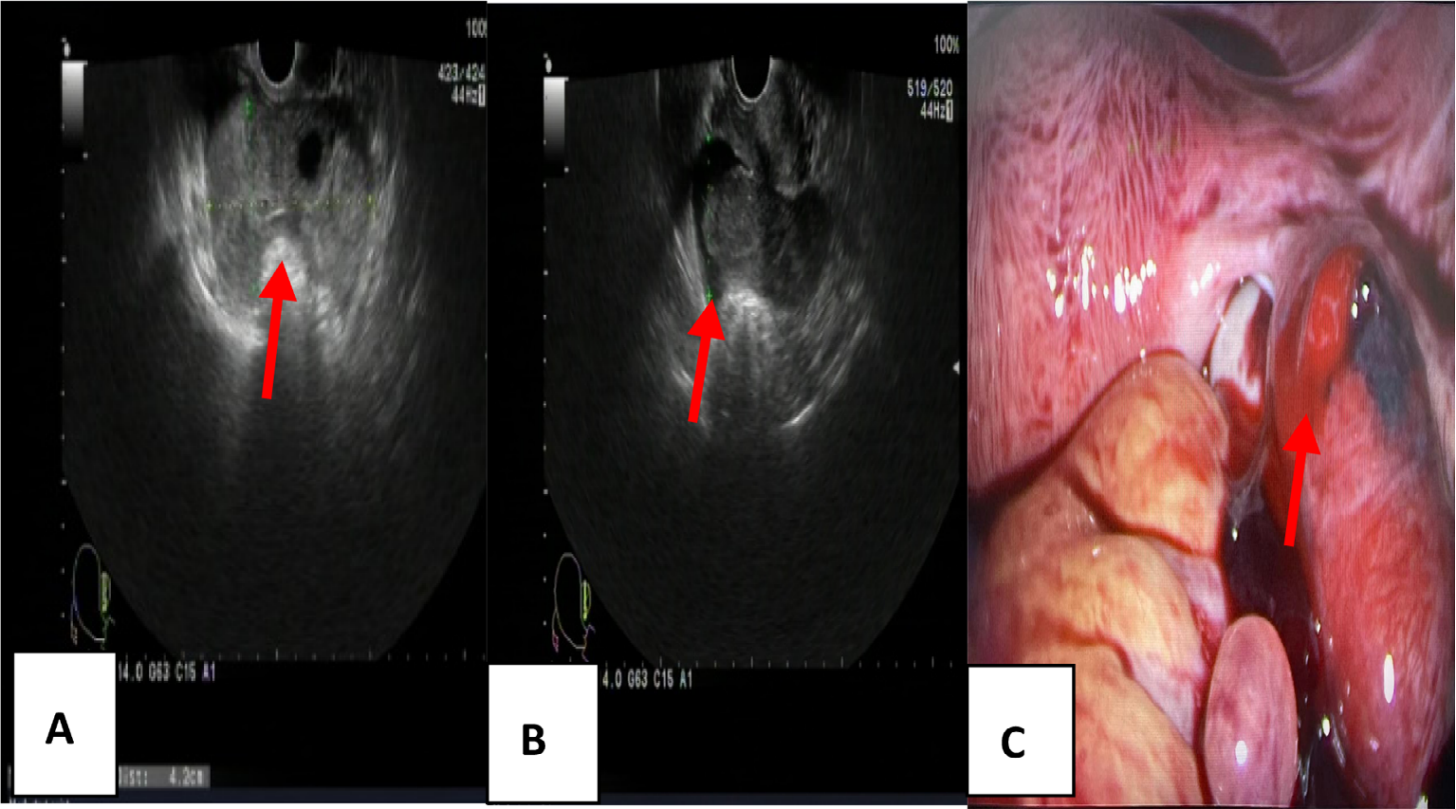
A 30‐year‐old women with RTP. **A**, Transverse sonogram showing a more mixed mass in the left fallopian tube; **B**, pelvic fluid volume; **C**, intraoperative photo displaying a visible rupture on the surface of the left fallopian tube.

Figures 1 and 2 show the ultrasound features of the 2 groups.

### Comparison of Ultrasound and Clinical Features between 2 Groups

**Figure 3:**
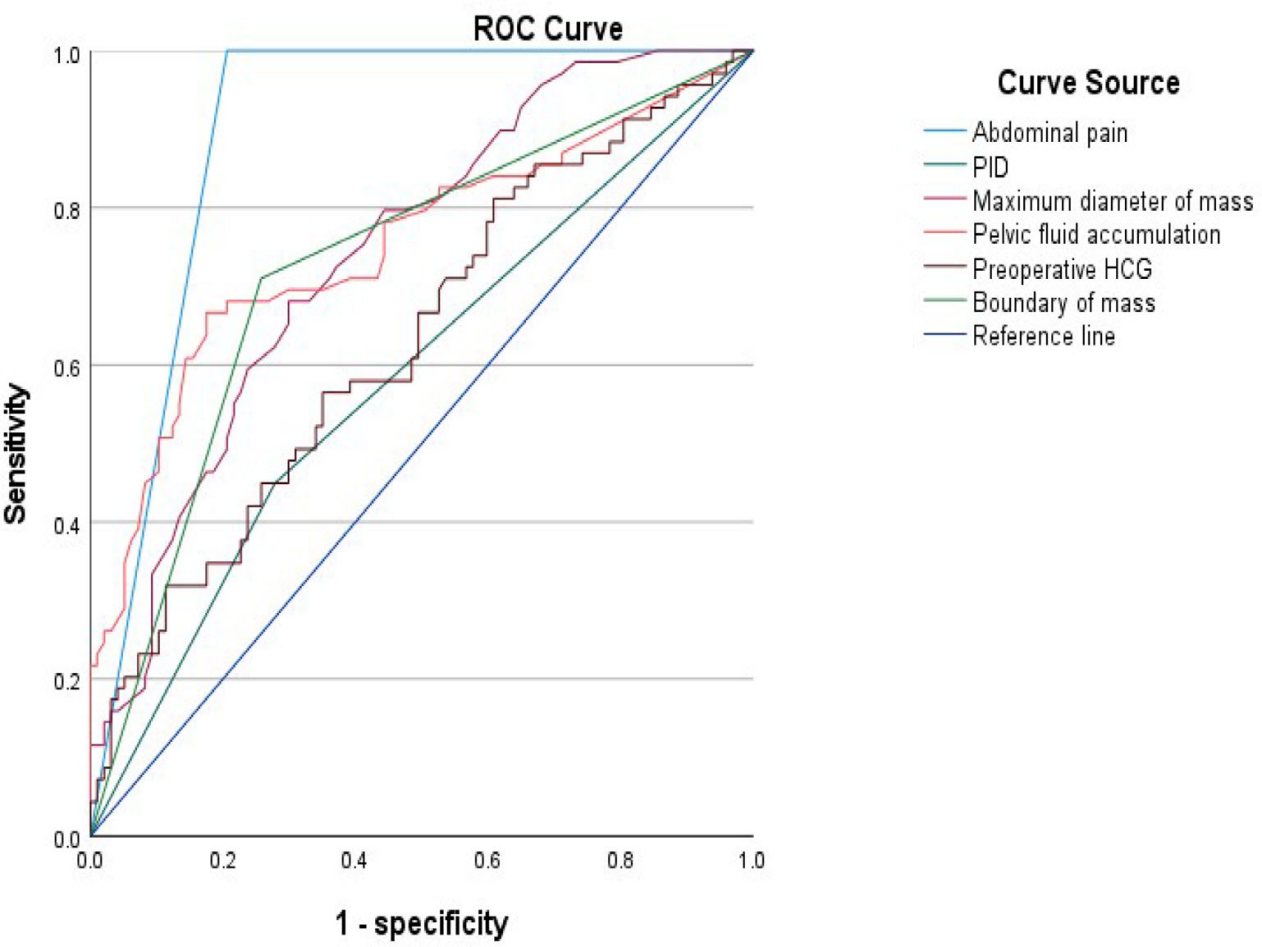
The ROC curve.

Univariate analysis showed that in the ultrasound features there was no difference between of uterine position, endometrial thickness, and mass location (*P* > .05), but the RTP group had a larger maximum mass diameter (50.86 ± 24.19 mm versus 32.99 ± 17.87 mm), unclear boundaries (71.01% versus 38.14%), and more pelvic fluid accumulation (45.14 ± 29.00 mm versus 20.46 ± 18.28 mm) (*P* < .05); In clinical features, there were no differences between of age, vaginal bleeding, intrauterine device, history of ectopic pregnancy, number of pregnancies, and history of abortion (*P* > .05). However, the RTP group had more cases of abdominal pain (100.00% versus 27.83%), pelvic inflammatory disease (14.49% versus 5.15%), high preoperative β‐HCG (10997.15 ± 19178.55 U/L versus 4639.97 ± 7243.70 U/L) (Table 1). ROC curve analysis showed that the maximum diameter of the mass, pelvic fluid accumulation, abdominal pain, preoperative β‐HCG could be used to evaluate tubal rupture, with AUC values of 0.741, 0.752, 0.861, 0.631 (*P* < .05); The AUC of 0.585 (*P* > .05) for pelvic inflammatory disease could not be used to evaluate the rupture of EP; The cut‐off values showed that the maximum diameter of the mass is >36.5 mm, the pelvic fluid accumulation is >34.5 mm, the preoperative β‐HCG is >3094.5 U/L, indicating the rupture of tube pregnancy(Figure 3 and Table 2). Multivariate logistic regression analysis showed that the pelvic fluid accumulation measured by ultrasound and preoperative β‐HCG were independent risk factors for tubal rupture (*P* < .05) (Table 3).

**Table 1:** Ultrasonic and Clinical Features Between 2 Groups

Parameter	UNRP (n = 97)	RP (n = 69)	*t*/*χ* ^2^	*P* value
**Ultrasonic features**				
Uterine position			2.903	.088
Anterior	69 (71.13)	64 (82.61)		
Posterior	28 (28.87)	5 (17.39)		
Endometrial thickness (mm)	9.63 ± 4.99	10.70 ± 5.01	−1.368	.173
Maximum diameter of mass (mm)	32.99 ± 17.87	50.86 ± 24.19	−5.482	.000
Location of mass			0.002	.961
Left fallopian tube	39 (40.21)	28 (40.58)		
Right fallopian tube	58 (59.79)	41 (59.42)		
Boundary of mass			33.403	.000
Clear or relatively clear	72 (74.23)	20 (28.99)		
Unclear	25 (25.77)	49 (71.01)		
Anecho in the mass			1.048	.306
Yes	34 (35.05)	19 (27.54)		
No	63 (64.95)	50 (72.46)		
Yolk sac			0.023	.879
Yes	16 (16.49)	12 (17.39)		
No	81 (83.51)	57 (82.61)		
Germ			1.497	.221
Yes	5 (5.15)	7 (10.14)		
No	92 (94.85)	62 (89.86)		
Fetal heart rate			0.767	.381
Yes	4 (4.12)	5 (7.25)		
No	93 (95.88)	64 (92.75)		
Pelvic fluid accumulation by ultrasound(mm)	20.46 ± 18.28	45.14 ± 29.00	−6.717	.000
**Clinical features**				
Age (years)	31.29 ± 5.45	31.91 ± 5.47	−0.726	.469
Days of amenorrhea (days)	46.22 ± 9.80	49.06 ± 12.78	−1.615	.108
Abdominal pain			102.161	.000
Yes	20 (20.62)	69 (100.00)		
No	77 (79.38)	0 (0.00)		
Vaginal bleeding			0.155	.693
Yes	69 (71.13)	51 (73.91)		
No	28 (28.87)	18 (26.09)		
Intrauterine device			0.174	.677
Yes	4 (4.12)	2 (2.90)		
No	93 (95.88)	67 (97.10)		
Number of pregnancy	2.04 ± 1.68	1.92 ± 1.7	0.424	.672
Number of abortion	1.22 ± 1.28	1.29 ± 1.44	0.346	.730
History of PID			5.182	.023
Yes	27 (27.84)	31 (44.93)		
No	70 (72.16)	38 (55.07)		
History of ectopic pregnancy			0.103	.748
Yes	6 (6.19)	5 (7.25)		
No	91 (93.81)	64 (92.75)		
Preoperative β‐HCG (U/L)	4639.97 ± 7243.70	10,997.15 ± 19,178.55	−2.984	.003

**Table 2:** ROC Analysis of Meaningful Factors of 2 Groups

Parameter	AUC	95% CI	*P*
Abdominal pain	0.897	0.847–0.947	.000
PID	0.585	0.497–0.674	.061
Maximum diameter of mass	0.741	0.667–0.815	.000
Boundary of mass	0.726	0.646–0.806	.000
Pelvic fluid accumulation	0.752	0.673–0.831	.000
Preoperative β‐HCG	0.631	0.545–0.717	.004

**Table 3:** Regression Analysis Results of Risk Factors Related to RP Group

Parameter	*β*	*P*	OR value	95%CI
Abdominal pain	47.022	.991	2.6E+20	
PID	−0.154	1.000	0.858	
Maximum diameter of mass	0.028	.300	1.029	0.975–1.085
Pelvic fluid accumulation	0.107	.007	1.113	1.030–1.202
Preoperative β‐HCG	0.000	.020	1.000	1.000–1.000
Boundary of mass	23.772	.997	2.1E+10	

## Discussion

This article was a study of 166 patients with tube ectopic pregnancy, including 97 cases of unruptured type and 69 cases of ruptured type. To search for quantitative indicators of ultrasound and clinical features that suggest tubal pregnancy rupture. The key results showed that the maximum diameter of the mass is >36.5 mm, the pelvic fluid accumulation by ultrasound is >34.5 mm, preoperative β‐HCG > 3094.5 U/L predicted the possibility of tube pregnancy rupture. Multivariate logistic regression analysis showed pelvic fluid accumulation by ultrasound and preoperative β‐HCG were independent risk factors for tube pregnancy rupture.

In the ultrasound features, the UNRTP group often had (28/97) circular mixed echogenic mass in the adnexal, the RTP group had a large mixed echo mass. There was no difference in the position of the mass, the anterior or posterior position of the uterus did not affect the implantation of the zygote in the fallopian tube (*P* > .05), and there was also no difference in the implantation of the zygote in the left or right fallopian tube. The endometrium showed decidual‐like reactions under the action of β‐HCG, so there was no difference in the endometrium thickness (10.70 ± 5.01 versus 9.63 ± 4.99 mm, *P* = .173). However, in the RTP group the maximum diameter of mass was larger (50.86 ± 24.19 mm versus 32.99 ± 17.87 mm, *P* = .000), and the boundary was unclear (71.01% versus 38.14%, *P* = .000), which may be caused by continuous erosion of the fallopian tube, outgrowth, and continuous bleeding due to high villous activity. ROC curve showed that the maximum diameter of the mass (95% CI 0.667–0.815, *P* = .000) could be used to evaluate the rupture of tubal pregnancy. The maximum diameter of mass >36.5 mm indicated the possibility of tubal rupture. Pelvic fluid accumulation the RTP group was more (45.14 ± 29.00 mm versus 20.46 ± 18.28 mm, *P* = .000), it may be caused by tubal rupture and hemorrhage accumulation into the pelvis, and the ROC curve showed that pelvic fluid accumulation could be used to evaluate tubal pregnancy rupture, and pelvic fluid accumulation >34.5 mm (AUC = 0.752, 95% CI = 0.673–0.831, *P* = .000) indicated the possibility of tubal rupture. In particular, multivariate regression analysis showed that the pelvic fluid accumulation measured by ultrasound (95% CI = 1.030–1.202, OR = 1.113, *P* = .007) was an independent risk factor for tubal pregnancy rupture.

In the clinical features, Previous studies^2^, ^3^ showed that the various risk factors for ectopic pregnancy include infertility,^4^ age >35 years,^7^, ^8^ previous ectopic pregnancy.^9^, ^10^, ^11^ However, in our study, age and previous history of ectopic pregnancy and previous abortions were not found to be risk factors for ruptured tubal pregnancy. Previous studies^12^, ^13^ had suggested that the longer the gestational age, the higher the risk of tubal pregnancy rupture. However, this study found no significant correlation between the duration of pregnancy (49.06 ± 12.78 versus 46.22 ± 9.80, *P* = .108) and tubal pregnancy rupture. Previous studies^9^, ^10^ had shown that the installation of intrauterine devices increased the incidence of tubal pregnancy, but there was no significant difference in the rupture of tubal pregnancy. As with this study, the intrauterine devices were not associated with tubal pregnancy rupture. Abdominal pain is one of the classic symptoms of tubal pregnancy.^9^ The cause of abdominal pain may be related to peritoneal irritation or bleeding due to tubal rupture.^13^ Consistent with previous research,^13^ the RTP group had a significantly higher incidence of abdominal pain (100.00% versus 27.83%, *P* = .000) compared to the UNRTP group. In the ROC curve analysis, abdominal pain (AUC = 0.897, 95% CI = 0.847–0947, *P* = .000) was used to evaluate the rupture of tubal pregnancy. Previous study^13^ had stated that abdominal pain is an independent risk factor for tubal pregnancy rupture in multiple regression analysis. However, in this study, abdominal pain was not an independent risk factor for tubal rupture (*P* = .991). PID can cause adhesions and narrowing of the fallopian tubes, obstructing the passage of fertilized eggs and leading to the occurrence of tubal pregnancy.^12^, ^13^, ^14^ Similar to the previous studies,^13^, ^14^ in this study, the RP group had more pelvic inflammatory diseases (14.49% versus 5.15%, *P* = .023). However, in the ROC curve, although the AUC of PID is 0.585 and the 95% CI is 0.497–0.674, *P* > .05, PID could not evaluate the rupture of tubal pregnancy. Previous studies^12^, ^13^ had showed that preoperative β‐HCG was related to tubal pregnancy rupture, and when preoperative β‐HCG was greater than 3000 U/L, it was related to tubal pregnancy rupture,^15^ but no specific values had been proposed. This study also found in the RP group the preoperative β‐HCG levels were significantly higher (10997.15 ± 19178.55 U/L versus 4639.97 ± 7243.70 U/L, *P* = .003). In the ROC curve, preoperative β‐HCG (AUC = 0.631, 95% CI = 0.545–0.717, *P* = .004) was used to evaluate the rupture of tubal pregnancy. This study also specifically concluded when preoperative β‐HCG > 3094.5 U/L suggested the possibility of tube pregnancy rupture. In multiple regression analysis, preoperative β‐HCG (95% CI = 1.000–1.000, OR = 1.000, *P* = .020) was showed to be an independent risk factor for tubal pregnancy rupture.

The accumulation of pelvic fluid measured by ultrasound and preoperative β‐HCG are independent risk factors for ruptured tubal pregnancy. A maximum mass diameter >36.5 mm, pelvic fluid accumulation >34.5 mm, preoperative β‐HCG >3094.5 U/L suggest a high likelihood of tubal rupture. This study provided specific quantitative indicators for the diagnosis of tubal pregnancy rupture, which is helpful for timely clinical treatment.

## Data Availability

The data that support the findings of this study are available on request from the corresponding author. The data are not publicly available due to privacy or ethical restrictions.
